# An Anxious Succession

**Published:** 1893-12-09

**Authors:** 


### The Hospital

An Institutional Journal of

Science, Medicine, Hygiene, Nursing, and News.

*For Hospitals, Asylums, Infirmaries, Dispensaries, Medical Practitioners, Students, and Nurses.*

Vol. XV.—No. 376.] [Dec. 9, 1893.

### An Anxious Succession.

A great physician, and something more, was the late Sir Andrew Clark. By the time that this is in the hands of readers Sir Andrew Clark's successor in the presidency of the Royal College of Physicians will have been elected. It is our hope that that successor may be a great physician, and something more. In the days, quite recent, when medicine was a mere art, based on no demonstrable science, the physician played the single part of more or less capable helper in the sick room. So far as the movements of the great world were concerned he was an outsider. But science has put the nurse into the physician's place in the sick room, whilst the physician himself has been transformed into a responsible scientific leader. This is a fact which cannot be denied, and it carries with it obligations which may not be repudiated.

Of the various factors which go to make up the complex sum of nineteenth-century intellectual life none is more vital, strenuous, and eager than the scientific factor. Science dominates the age. We converse, we worship, we even woo in terms of science. Of sciences and scientists themselves none are more watchful, more ardent, more resolutely progressive than the sciences and scientists which concern themselves with medicine. One inevitable result of all this is that medicine no longer plays a merely domestic and sick-room part among mankind. On the contrary, it is one of the most conspicuous and most keenly observed of all the players which figure in this nineteenth century on the stage of the greater world. All these things are occasions of gentle regret to some of those valetudinarian physicians who, as is the fashion of old age, think "the former days were better than these." A sympathetic tear may be dropped upon the closed grave of the past, even by those who are yet in the full vigour of life. But every man who would play a man's part in the present must steel his nerves, and remembering that the past is past, and can return no more, must meet modern times and modern men with modern methods and a modern mind.

The president of the Royal Society for the time being is a great personage in both the scientific and the outer worlds. He is one of the kings of modern times, much more regal, much more potent than any but the greatest kings of the present or the past. "Thousands wait upon his nod," to quote Burns. To tens of thousands he is an oracle. Similarly the president of the British Association in any given year becomes a conspicuous figure throughout the civilised world by the mere fact of his presidency. The world at once admits his claims to distinction. The utterances of men who speak from these elevations sound out and are echoed and carried to the remotest regions where civilised man finds a resting place. These are significant facts. They remind us how necessary it is that the presidents of such associations should not be weak men, should not be dumb dogs which cannot bark, should not in fact be anything but men of intellectual might, of majestic character, and of utterance so high and noble that all the world may profit by the hearing of it. Has the great world as keen an interest in the personality of the president of the Royal College of Physicians as in that of the president of the Royal Society? In former years, until quite recently indeed, this question must have been answered in the negative. Nobody knew and nobody cared who might be the president of the Royal College of Physicians. But all this has been to a large extent changed. During Sir Andrew Clark's incumbency the president of the Royal College of Physicians was as widely known, and even more widely interesting to the public than the president of the Royal Society himself. The position thus assuredly gained for the College of Physicians is a gain of great significance to the whole medical world and also to the public at large. This position must be maintained, and it must be used as a stepping stone from which Medicine and the College of Physicians may rise to yet higher and higher positions of public honour and public usefulness.

Sir Andrew Clark made the presidency of the English College of Physicians famous throughout the world. How did he do it? He did it because he could not help himself. At the commencement of his presidency he felt himself, like previous presidents, bound, swathed, and imprisoned by written and unwritten traditions; and he was minded, if possible, to preserve those traditions unimpaired. But it was not possible. So far as we know he committed no single act which shocked those traditions, even in the slightest degree. But his whole character, his mind, and his sympathies constituted one long protest against narrowness, secrecy, and professionalism in every form. Without even once saying that the modern lion ought no longer to be bound by the net of a narrow and unworthy traditionalism, he came in time to feel that the net was breaking of itself, and that he and all the other robust-minded Fellows of the College would soon find themselves as free to breathe and move and act on the stage of the widest public life as if they had never been bound by the narrow fellowship of one of the narrowest corporations in the world.

No future president of the College of Physicians can ever be unaware of the fact that he is the foremost figure among doctors, and that by him medicine is represented to his fellow-countrymen. Every variety of medical and surgical practice and of medical and surgical practitioner is for practical and public purposes concentrated in his single personality. In him the unknowing world will see medicine and her representatives; and by him medicine and her representatives will be raised to honour, or sunk to mediocrity and obscurity. The personality of the president of the English College of Physicians is for English practitioners of all ranks the most important of all public facts and personalities. So much is this the case, indeed, that we do not hesitate to affirm that the only really satisfactory method of electing such a personality would be that of universal medical suffrage.

For all future time the president of the Royal College of Physicians must be a public man. It is his *rôle* to be public and to be looked at. If he will be public he will be public, and if he will not be public he must be public. He cannot help himself. The times and the movements of the times will have it so. This inevitableness of things the Fellows of the College of Physicians must recognize, and they must give their president the charter of a wide and generous freedom for every variety of public and representative duty. It is certain that no man whom the College in its discretion may elect will ever abuse such freedom, or use it for personal ends. There is a wide range of choice among venerable seniors of proved character and life, and if among such seniors not one can be found who is worthy of the most unlimited freedom, then the College should forthwith prepare to ring its own death-knell as a failure unworthy of survival in an age of intellectual liberty. As for those few suspicious fellows "of the baser sort," who think no man is to be entrusted with unlimited freedom, let them be reduced to silence and obscurity by the unuttered scorn of all right-minded men.

For ourselves, and we are sure we speak the sentiments of the vast majority of our fellow-practitioners, we desire to see the president of the Royal College of Physicians play a great and honourable part among the leaders of opinion and action. His natural equals are the Archbishop of Canterbury, the Lord Chancellor, the President of the Royal Society, the heads of the Church, the Law, and Commerce, and all who play active and significant parts in the public life of the nation. In the leadership also of those who charge themselves with the nation's health, and with the care and cure of the sick of all ranks, what various and important duties stand revealed! The president of the College of Physicians is not head of the College of Physicians merely, but head and king of the medical profession—of hospitals, of nursing, and of all that pertains to the sick—a position immeasurably higher. The medical profession and the sick world looks with an anxious and sympathetic heart towards its new leader. Let him but be a man, let him but claim and use the freedom without which no human creature, however variously accomplished, can be a great man; let him but be a worthy successor of the magnanimous and gentle spirit which has just passed away, and the whole profession of medicine, with the whole world of sickness, will give unstinted gratitude to the Fellows of the College of Physicians who have put him in his honourable place.

